# Effects of a snack on performance and errors during a simulated 16-h night shift: A randomized, crossover-controlled, pilot study

**DOI:** 10.1371/journal.pone.0258569

**Published:** 2021-10-22

**Authors:** Sanae Oriyama, Kotomi Yamashita

**Affiliations:** 1 Division of Nursing Science Graduate School of Biomedical and Health Sciences, Hiroshima University, Hiroshima, Japan; 2 Doctoral Program in Biomedical and Health Sciences, Hiroshima University, Hiroshima, Japan; University of Lübeck: Universitat zu Lubeck, GERMANY

## Abstract

**Background:**

Night shift workers might not eat due to their busy schedules during the night shift. However, food may not only satisfy hunger, but also affect performance and errors. The aim of this study was to clarify the effect of a snack on performance and errors during 2-day, 16-h, simulated night shifts.

**Methods:**

A randomized, repeated-measure, crossover study was performed to investigate subjective and cognitive performance in 15 healthy female adults (mean age, 21.7 years) after they consumed a snack (352 kcal) during a simulated night shift (16:00 to 09:00) from October to November 2018. The participants were kept awake from waking up in the morning to the next day at 09:00. Subjects were tested for performance on the Uchida-Kraepelin test, as well as for subjective feeling, body temperature, psychomotor vigilance test, and heart rate variability, before and after they consumed the snack. One day before the experiment, all participants wore an actigraphy monitoring device to determine their sleep state.

**Results:**

There was no difference between having (Snack condition) and not having (Skipping condition) the snack in sleep states the day before the experiment. On the day of the experiment, between 16:00 and 09:00, subjective sleepiness, fatigue, and body temperature were not different between the two conditions. Subjects maintained performance on the Uchida-Kraepelin test and showed a significant improvement in false starts on the psychomotor vigilance test, the primary outcome measure, in the Snack condition compared with the Skipping condition. The Snack condition was also associated with decreased high-frequency power, a decreased low-frequency power/high-frequency power ratio, and increased heart rate in the vagally mediated heart rate variability indices, which may reflect a higher ability to modulate cognitive and behavioral processes.

**Conclusions:**

These results suggest that providing a snack to shift workers during night shifts might improve work safety and efficiency.

## Introduction

As Japan is increasingly becoming a 24-h society [1], rotating shifts have come to incorporate nighttime work, which has led to the necessity of shift work in a variety of service sectors. However, compared with the day shift, shift work can pose threats to the health of workers and increase the risk of developing various health problems. Despite these risks, the number of shift workers in Japan has continued to rise [1]. The healthcare professions in Japan, especially nurses, represent a typical example of an industry that has increasingly adopted a shift work system. Since 2017, the number of nurses working 16-h night shifts in Japan has increased significantly [2], which has resulted in increased physical and mental burdens. Nurses working on such schedules often experience morning sleepiness and fatigue [3, 4]. In addition, it has been reported that long working hours are associated with a higher risk of errors and accidents and poorer performance [5]. Compared with the traditional 8-h working day, the risk of occupational injury when working more than 12 h per day is estimated to be 147% higher [6].

The activities of many organisms on earth have a circadian rhythm with a cycle of about 24 h, the same as the rotation of earth. In addition, the activities of the endocrine system and nervous system are synchronized with activity during the day and the rhythm of rest and sleep at night [7]. The source of the circadian rhythm is the biological clock that gives periodicity to the biological mechanism by endogenous autonomous vibration, and the main clock located in the suprachiasmatic nucleus (SCN) of the hypothalamus acts as a master circadian pacemaker, regulating other (peripheral) clocks throughout the body and thus maintaining physiological rhythmicity [8]. In this way, the function of the body fluctuates at a certain cycle throughout the day. For example, it is known that the body temperature peaks in the time period from 19:00 to 21:00 and becomes the lowest in the middle of the night from 02:00 to 04:00. Since there is a close relationship between arousal level and body temperature, activity during times of low body temperature imposes a burden on the body itself. On the other hand, during the daytime after a night shift, the sleep quality is lower in the time period when the body temperature is high than during nighttime sleep. Such deviations can lead to various health problems triggered by overwork and insomnia. Night work has been reported to increase morning sleepiness because of disruptions in circadian rhythms [9].

In addition, regarding dietary patterns during night shifts, avoiding eating altogether has been investigated as a strategy to maintain alertness [10]. A previous study [11] investigating the effects of food intake during nighttime (from 01:30 to 02:30) found that fasting resulted in reduced performance in a cognitive vigilance task. Another study reported that eating a snack during the night shift (at 01:30) alleviated hunger without leading to perceived fullness or increased sleepiness until 05:00 [12], although the snack’s effect duration has remained unknown. Portion size is an important aspect of nighttime eating. Among nurses, reduced meal sizes have been reported as a strategy for minimizing gastric symptoms during night shifts [10]. However, to the best of our knowledge, the duration of the effects of food intake and the potential impact on performance have not been reported in previous studies investigating the effects of sleep loss on performance. Given this background and the fact that performance is most severely impaired at night, reducing food intake during night shifts may serve as a suitable countermeasure. If the duration of the snack effect could be clarified, it could help answer the core questions of both what and when night shift workers should eat to avoid metabolic disturbances; this, in turn, could optimize wakefulness and performance and help prevent accidents in the workplace. Accordingly, in this study, a rice ball was selected as a midnight snack that night shift workers can easily eat even if they are busy. Rice is a staple food for more than half of the world’s population. In addition to calories, rice is a good source of magnesium, phosphorus, manganese, selenium, iron, folic acid, thiamin, and niacin, but it is low in fiber and fat.

Therefore, this study aimed to investigate the impact of eating (the Snack condition) compared with not eating (the Skipping condition) at night on performance, sleepiness, fatigue, and other physiological measures during 16-h simulated night shifts. We hypothesized that, under the conditions of extended wakefulness during the “biological night,” eating a snack would lead to decreased errors and maintain performance until the morning. To test this hypothesis, sleepiness, hunger, vigilant attention, cognitive processing, and other physiological measures were assessed under both Snack and Skipping conditions during consecutive, 2-day, 16-h, simulated shifts throughout the night.

## Materials and methods

### Study design and participants

**Fig 1:**
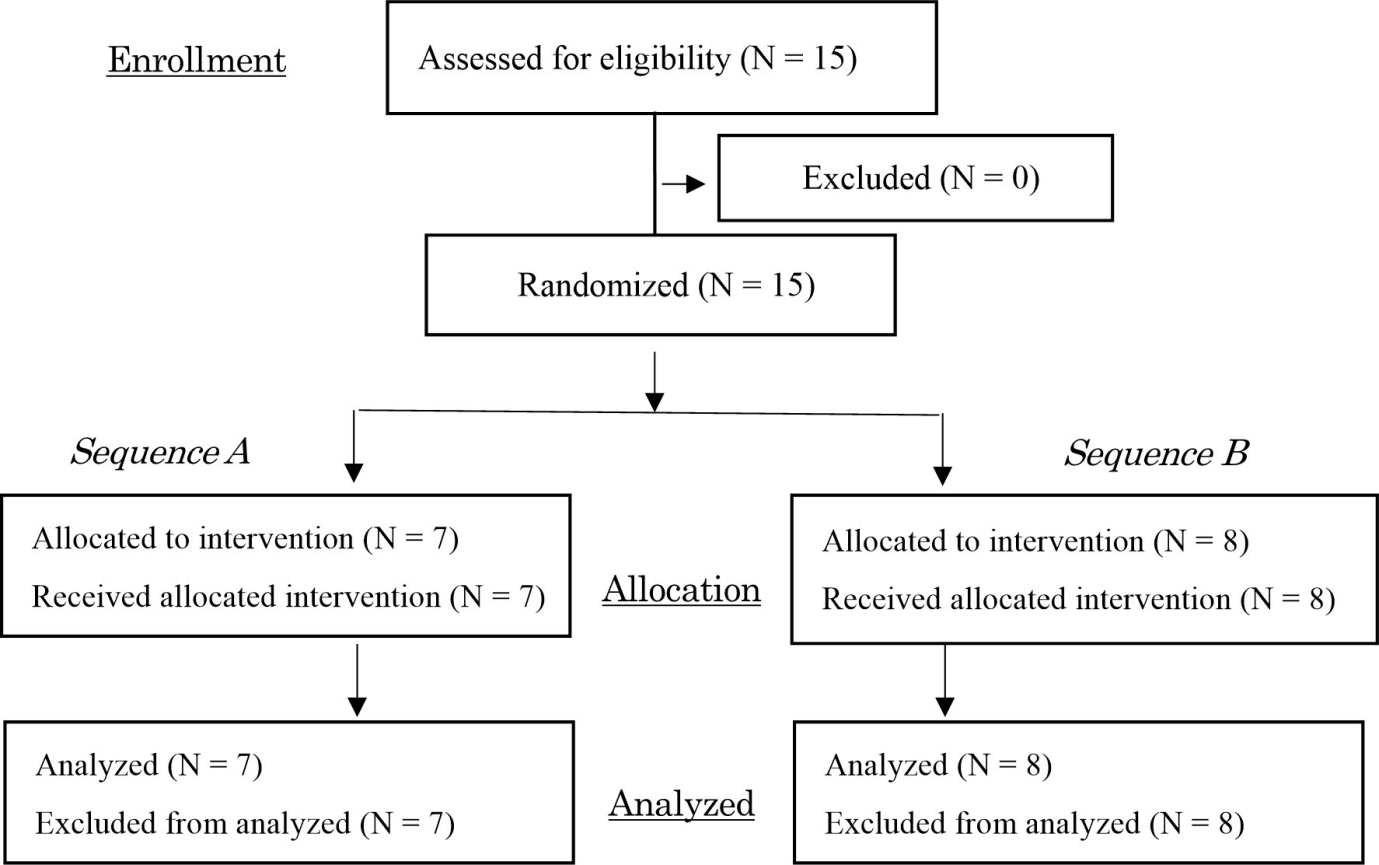
Flowchart of the study design.

The participants in this randomized, crossover study were 15 healthy adult females (mean age ± standard deviation [SD], 21.7 ± 0.5 years; body mass index, 19.81 ± 2.45 kg/m^2^) recruited between October and November 2018. None of the subjects had any previous night shift experience, and none was identified as morning type or evening type according to the morningness–eveningness questionnaire [13]. All of the participants were current nonsmokers, in the luteal phase of their menstrual cycle [14, 15], and had normal sleep patterns (habitual sleep ranging between 7 and 9 h). No participants were currently taking any prescribed medications. The study flowchart is shown in Fig 1. All participants were randomly allocated to either sequence A or B using random numbers generated by Excel. The required sample size was determined to be 14 (actual power 81.0%) based on an effect size, α error, and power (1-β) of 0.25, 0.05, and 0.8, respectively. The power calculation in this study was carried out using G*Power 3 [16].

### Study meal

All participants consumed one or two meals between 16:00 and 09:00. Under both the Snack and Skipping conditions, all participants ate a meal (containing 708 kcal, 19.4 g protein, 17.9 g fat, 112.4 g carbohydrates, and 3.5 g sodium) at 19:30 (not eating at night), and under the Snack condition only, participants ate a snack at 03:30 (eating at night). The meal provided at 03:30 consisted of two rice balls (containing 352 kcal, 5.8 g protein, 1.8 g fat, 75.8 g carbohydrates, and 1 g sodium) and two slices of yellow pickled radish. Many night shift workers prefer a high-energy diet rich in carbohydrates [17], such as the rice balls that are widely available at any convenience store in Japan. All participants were given 20 min to finish their meals, and they were encouraged to eat everything on their plate.

### Study protocol

**Fig 2:**
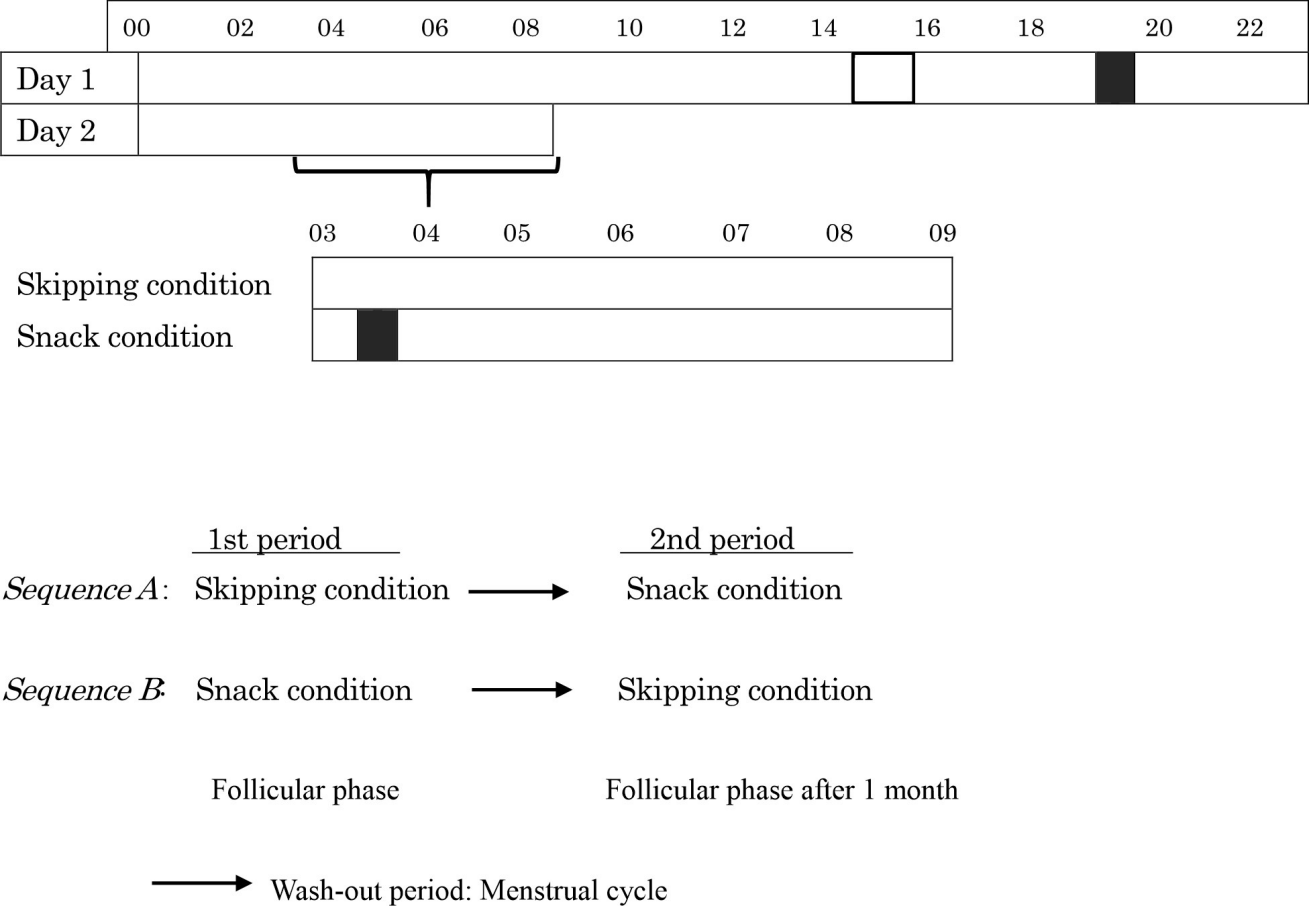
Schematic of the study protocol and experimental schedule. In the protocol, each row represents 24 h. The black area indicates eating time. The dotted area indicates the time of practice measurements. The diagonal hatching shows both conditions during that time period. In the experimental schedule, there are the two groups. Black arrows indicate the menstrual cycle period.

The protocol and experimental schedule of this study are shown in Fig 2. The measurements were conducted over 2 consecutive days between 16:00 and 09:00. Each experiment day had 5 participants who were randomly assigned to one of two conditions using counterbalancing. All participants were instructed to refrain from strenuous physical exercise and not to consume caffeine or alcohol for 24 h prior to and during each study day. Two days before the experiment began, all participants wore an actigraphy monitoring device (ActiGraph; Ambulatory Monitoring Inc., Ardsley, NY, USA) on their non-dominant wrist and recorded their sleep and activity levels in a diary.

On the day of the experiment, all participants arrived at the laboratory at 15:00. Until 16:00, they carried out practice assessments, including the Uchida-Kraepelin test (UKT) and the psychomotor vigilance test (PVT). The Snack condition involved consuming a meal and a snack at 19:30 and 03:30, respectively, whereas the Skipping condition involved consuming a meal at 19:30 only. At the start of each experiment, the participants were fitted with a heart rate variability (HRV) sensor (GMS Inc., Tokyo, Japan). For each hour throughout the experiment, they were given 10 min to record their sublingual temperature once and complete the visual analog scale for sleepiness, fatigue, and hunger, 10 min to perform the UKT, and 10 min to measure the PVT. The next 20 min were considered free time, and the remaining 10 min were considered a rest period. The participants spent their free time reading, drawing, or drinking water. During the 10-min rest period, they sat on chairs and chatted with the other participants. The same meal amounts and contents were given to the participants between 19:30 and 19:50 in each experimental period. The HRV sensor was removed at the end of each experiment, but the participants were asked to continue wearing the actigraphy monitoring device until they woke up the next day. During all waking times, the participants remained awake in the laboratory and were continuously monitored by the researchers.

All participants stayed in a windowless and sound-insulated laboratory for 2 consecutive days (1 night). The laboratory environment was maintained at 26 ± 2°C [18] and 50% relative humidity under indoor illumination on the table at 200 lx.

### Measurements

#### Sleep parameters

The sleep parameters measured were total sleep time, sleep efficiency (total sleep time / time in bed × 100), sleep onset latency, and wake after sleep onset. All parameters were measured using the actigraphy monitoring device, and they were analyzed using the AW2 software package (Ambulatory Monitoring Inc.).

#### Physiological parameters

The circadian rhythm of body temperature is one of the most frequently used indicators of circadian rhythmicity [19], and body temperature has been shown to be related to sleepiness, fatigue, and performance of a single-digit mental arithmetic task [20]. Sublingual temperature, which is considered an index of internal body temperature [21], was measured hourly using an oral thermometer (MC-612; Omron Inc., Kyoto, Japan) to assess changes in circadian modulation during the night.

#### Cognitive performance

A visual analog scale was used for the subjective assessment of sleepiness, fatigue, and hunger [22]. The participants rated their sleepiness, fatigue, and hunger every hour on a 100-mm line, with values ranging from 0 mm (not sleepy, tired, or hungry at all) to 100 mm (extremely sleepy, tired, or hungry).

The UKT (Nisseiken, Tokyo, Japan), a serial mental arithmetic task, was used to measure cognitive performance. This test is a questionnaire that requires intense concentration and effort, and it has been used as a tool to induce mental stress [23]. The test material consisted of a pre-printed paper with 20 rows of 115 random, single-digit figures. The subjects’ task was to add adjacent figures horizontally, and then write the one-digit answer between the 2 figures; they were asked to proceed along each row as quickly and as accurately as they could in a 1-min period. On being given the first cue, the subjects began calculating from the first row. Then, when the second cue was given after 1 min, the subjects were required to begin a new row, without regard to their position on the current row. This procedure was repeated 8 more times, for the total duration of 10 min. The sum of the correct answers and errors for each 1-min period over the 10-min task was used as the value for the analysis.

The PVT is a reaction time task considered to be a sensitive measure for assessing the effects of sleep loss [24]. In this study, a precise computer-based version of the 10-min PVT was used to avoid problems of uncertainty with regard to the accuracy of the test platform timing [25]. All participants were instructed to look at a computer monitor and press a response button when a white circular edge appeared on the screen; pressing the response button stopped the counter and displayed the response time (in milliseconds) for a 1-s period. The PVT measures response times to visual stimuli randomly occurring at 2- to 10-s intervals over a 10-min period [25]. The outcome measures for the PVT included the median response time, number of lapses (response time > 500 ms), and false starts (defined as any response in the absence of a stimulus or within the first 100 ms of stimulus responses: time < 100 ms) [26, 27], as well as mean response time.

#### Autonomic nervous system activity

For the purposes of the present study, HRV was obtained through autoregressive analysis of R–R intervals measured between 16:00 and 09:00. All data were analyzed offline after analog-to-digital conversion of 250-Hz R–R waves. HRV was measured every 5 min during each hour and then averaged; these measurements were used to monitor autonomic nervous system activity throughout the night [28]. High-frequency (HF) and the low-frequency/high-frequency (LF/HF) ratio are used as indicators of cardiac parasympathetic and cardiac sympathetic nervous activity, respectively [29, 30]. The LF/HF power ratio indicates the balance between sympathetic and parasympathetic outflows [31].

### Statistical analysis

All results are shown as means ± SD or standard error of the mean. All sleep variables measured the day before the experiment were analyzed using the *t*-test.

To test the effects of consuming a snack on neurobehavioral and physiological outcomes during the early morning measurement periods, a fully saturated, linear mixed-effects analysis of variance was carried out [32], with a between-participant fixed effect of condition and a within-participant fixed effect of time (at 03:00 vs. from 04:00 to 09:00) and a random intercept. Within-condition comparisons were used to minimize the effect of individual differences. Multiple comparisons were assessed using the Bonferroni correction to evaluate patterns of change under the two conditions. As a secondary analysis, between-condition hourly comparisons from 16:00 to 09:00 were analyzed using the Mann-Whitney U test.

To assess the postprandial effect of the meal throughout the testing time, the net incremental area under the curve (niAUC), calculated from pre- (at 03:00) and postprandial time points, was tested.

Correlations were used to assess the relationships of performance with temperature and autonomic nervous system activity. The performance outcome measure used in the analysis was the change from after eating (04:00 to 09:00). The performance variables of interest were PVT and UKT.

All statistical analyses were conducted using SPSS (version 22.0J; IBM, Tokyo, Japan). The hypothesis rejection level for all tests was set at *p* < .05, and a notable trend was set at *p* < .1.

### Ethical considerations

This study was approved by the Center for Integrated Medical Research of Hiroshima University (approval number: C-252) and the University Hospital Medical Information Network (UMIN)-Clinical Trials Registry (CTR) in the Japan Primary Registries Network (JPRN) registry (approval number: UMIN 000034345; approved on October 1, 2018). Written, informed consent was obtained from all participants before the first examination. The study protocol conformed to the Declaration of Helsinki guidelines. The authors confirm that all ongoing and related trials for this intervention have been registered.

## Results

### Sleep state before the experiment

Total sleep time, sleep efficiency, sleep onset latency, go to bed time, and wake up time, assessed by the ActiGraph, were not different between phases (See Table 1).

**Table 1:** Sleep sensitivity parameters by meal condition.

Variable	Snack	Skipping	p-value
ActiGraph			
Total sleep time, min	461.9 (65.1)	470.5 (73.4)	.735
Sleep efficiency, %	93.6 (5.4)	94.5 (6.0)	.663
Sleep onset latency, min	10.9 (33.3)	7.5 (16.7)	.732
Go to bed time, h:min	00:30 (01:21)	23:54 (01:07)	.562
Wake up time, h:min	08:26 (01:21)	07:57 (01:04)	.288

Date are means (SD), N = 15.

### Physiological parameters

**Fig 3:**
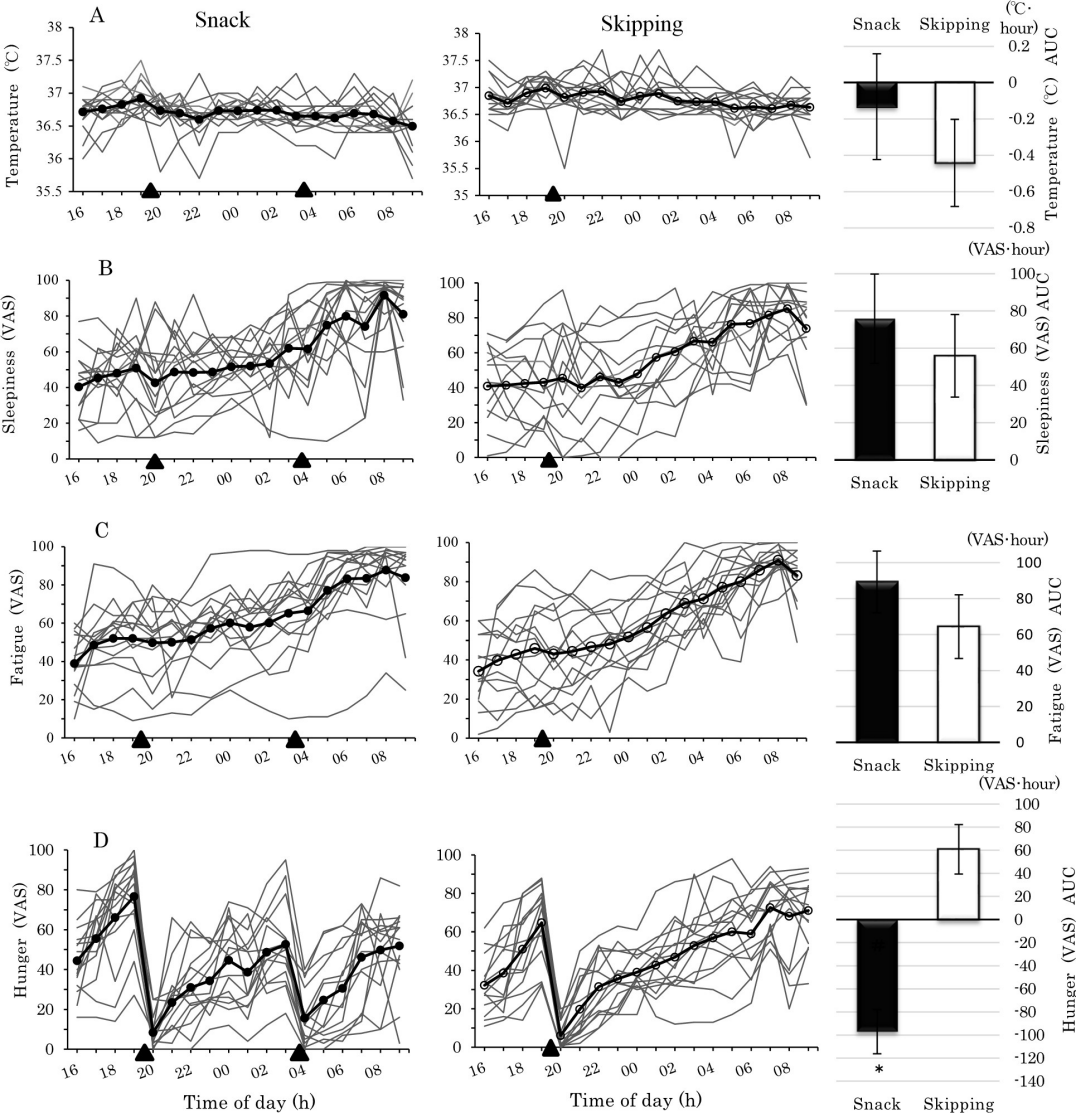
Temperature, sleepiness, fatigue, and hunger following a simulated night shift. A-Temperature, B-Sleepiness (VAS: Visual Analog Scale), C-Fatigue (VAS), D-Hunger (VAS). Left side and center panels display the individual data per condition from 16:00 to 09:00 testing points. Snack condition—left side panel, Skipping condition—center panel. The means are shown over the top of the individual data. Both conditions are thick solid black lines, the Snack condition is shown with a black circle, and the Skipping condition is shown with an open circle. Black triangle (▲) indicates eating a meal at 19:30 and at 03:30. The average values of the markers in the left side and center panels match columns in the right side panels for each condition, before and after eating, from 03:00 to 09:00. Bars represent standard error. Analysis of covariance was used to assess the significance of mean differences between groups after eating (*: p < .05).

**Fig 4:**
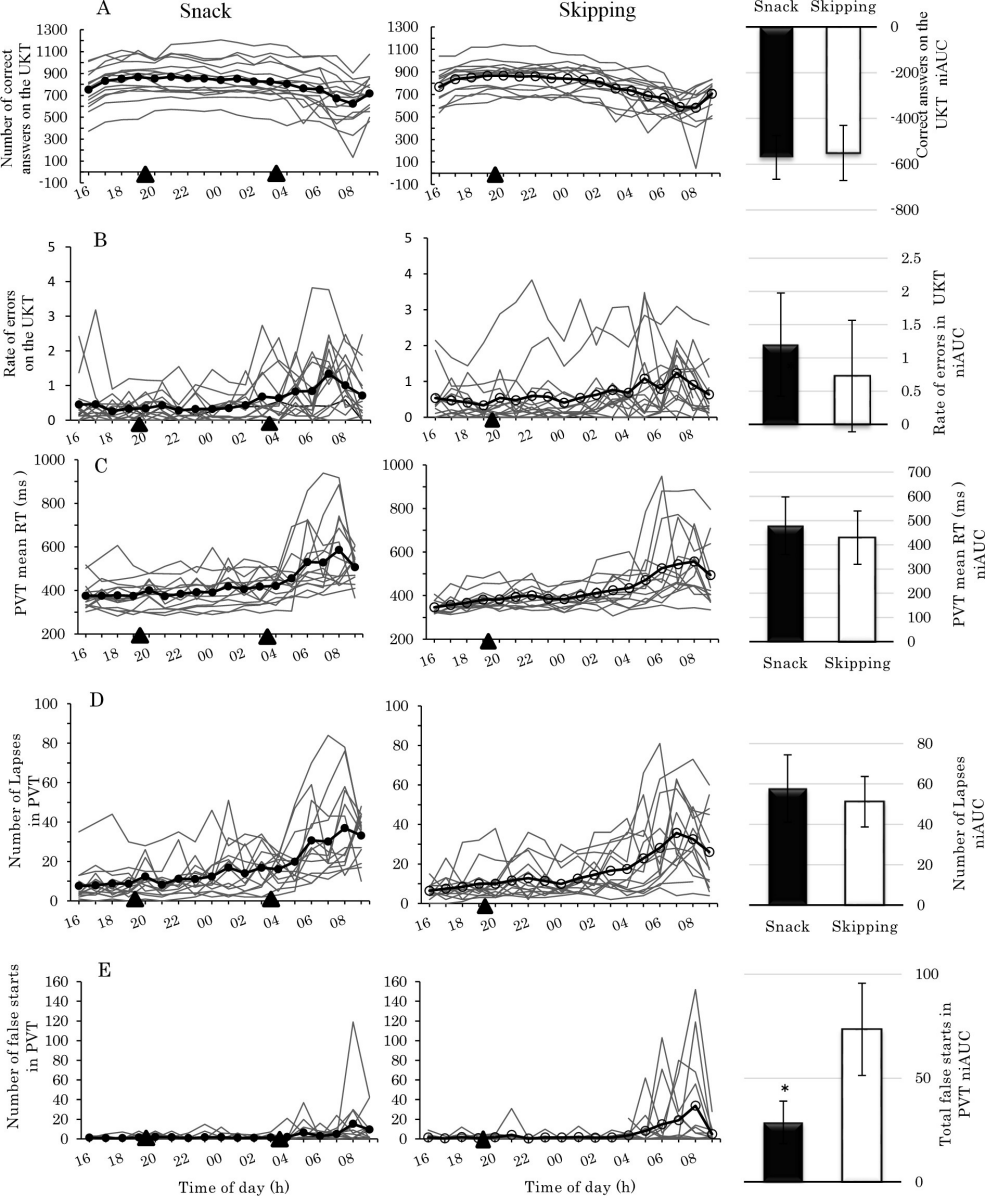
Uchida-Kraepelin Test and Psychomotor Vigilance Task following simulated night shift. A-Uchida-Kraepelin Test, B-Rate of errors on the Uchida-Kraepelin Test, C-Psychomotor Vigilance Task mean RT, D—Psychomotor Vigilance Task lapses, E-Psychomotor Vigilance Task false starts. Left side and center panels display the individual data per condition from 16:00 to 09:00 testing points. The means are shown over the top of the individual data. Both conditions are shown as thick solid black lines, the Snack condition is shown with a black circle, and the Skipping condition is shown with an open circle. Snack condition—left side panel, Skipping condition—center panel. Black triangle (▲) indicates eating a meal at 19:30 and at 03:30. The average values of the markers in the left side and center panels match columns in the right side panels for each condition, pre- and after eating, from 03:00 to 09:00. Bars represent standard error. Analysis of covariance was used to assess the significance of mean differences between groups after eating (*: p < .05).

Outcomes regarding the participants’ physiological parameters, cognitive performance, and autonomic nervous system activity from 16:00 to 09:00 are shown in Figs 3 and 4. From 16:00 to 03:00, no significant main effect of their interaction was observed, and the analysis from 03:00 to 09:00 is shown in Table 2.

**Table 2:** Linear mixed models: Physiological parameters, cognitive performance, and autonomic nervous system activity outcome measures.

Outcome Measure	Condition		Time		Condition*time	
	F df	p	F df	p	F df	p
**Physiological parameter**						
Temperature	1.690 _1,182_	.195	1.297 _6,182_	.260	1.004 _6,182_	.424
**Cognitive performance**						
Subjective sleepiness	.166 _1,182_	.684	6.246 _6,182_	**< .001**	.688 _6,182_	.659
Fatigue	.592 _1,182_	.443	11.469 _6,182_	**< .001**	.332 _6,182_	.919
Hunger	135.198 _1,182_	**< .001**	12.763 _6,182_	**< .001**	5.898 _6,182_	**< .001**
UKT (Total number)	16.124 _1,182_	**< .001**	10.950 _6,182_	**< .001**	.536 _6,182_	.780
UKT (Rate of errors)	.216 _1,182_	.643	2.590 _6,182_	**.020**	.407 _6,182_	.873
PVT (Mean RT)	.005 _1,182_	.942	6.591 _6,182_	**< .001**	.154 _6,182_	.988
PVT (Median RT)	.201 _1,182_	.655	5.516 _6,182_	**< .001**	.354 _6,182_	.907
PVT (Lapse)	.116 _1,182_	.734	6.764 _6,182_	**< .001**	.576 _6,182_	.749
PVT (False starts)	4.420 _1,182_	**.037**	4.867 _6,182_	**< .001**	1.294 _6,182_	.262
**Autonomic nervous system parameter**						
HR	32.036 _1,169_	**< .001**	1.816 _1,169_	.099	2.411 _6,169_	**.029**
LF/HF	1.045 _1,169_	.308	3.068 _6,169_	**.007**	1.914 _6,169_	.081
HF	11.930 _1,169_	**.001**	1.667 _6,169_	.132	1.016 _6,169_	.417

Results shown are from linear mixed model analyses with main effects of condition: Snack condition/Skipping condition; time from 03:00 to 09:00 (interval between 1 h) and their interactions (condition*time). UKT: Uchida- Kraepelin Test; PVT: Psychomotor Vigilance Test; HR: heart rate; LF/HF: low-frequency power/high-frequency power ratio; HF: high-frequency power, bold indicates a significant value (p < .05), F-statistic: variation between sample means, df: degrees of freedom.

### Cognitive performance

The interaction between condition and time of measure was not significant for sleepiness and fatigue, such that sleepiness and fatigue increased in the two conditions from 03:00 to 09:00; a similar trend was seen across the night to early morning (See Fig 3B and 3C, left, center panel).

The interaction between eating condition and time of measurement was significant for hunger (*p* < .001; Table 2). As can be seen in Fig 3D (left, center panel), hunger decreased in the Snack condition compared to the Skipping condition (*p* < .001) from 04:00 to 09:00. In Fig 3, at the start of the experiment, sleepiness, fatigue, and hunger differed, with VAS values from 10 to 80 for individuals.

The results of the correct answers and the rate of errors on the UKT are shown in Fig 4A and 4B. Significant main effects of condition were observed for the number of correct answers, in which the number of correct answers achieved from 04:00 to 09:00 was significantly better in the Snack condition than in the Skipping condition (*p* < .001).

### PVT

Results regarding the mean RT, lapse, and false starts on the PVT from 03:00 to 09:00 are shown in Fig 4C–4E. On PVT, mean RT, and lapse, there were no significant main or interaction effects. On the other hand, false starts showed a significant main effect of condition (*p* = .037); false starts were significantly fewer under the Snack condition than under the Skipping condition (Fig 4E).

### Autonomic nervous system activity

**Fig 5:**
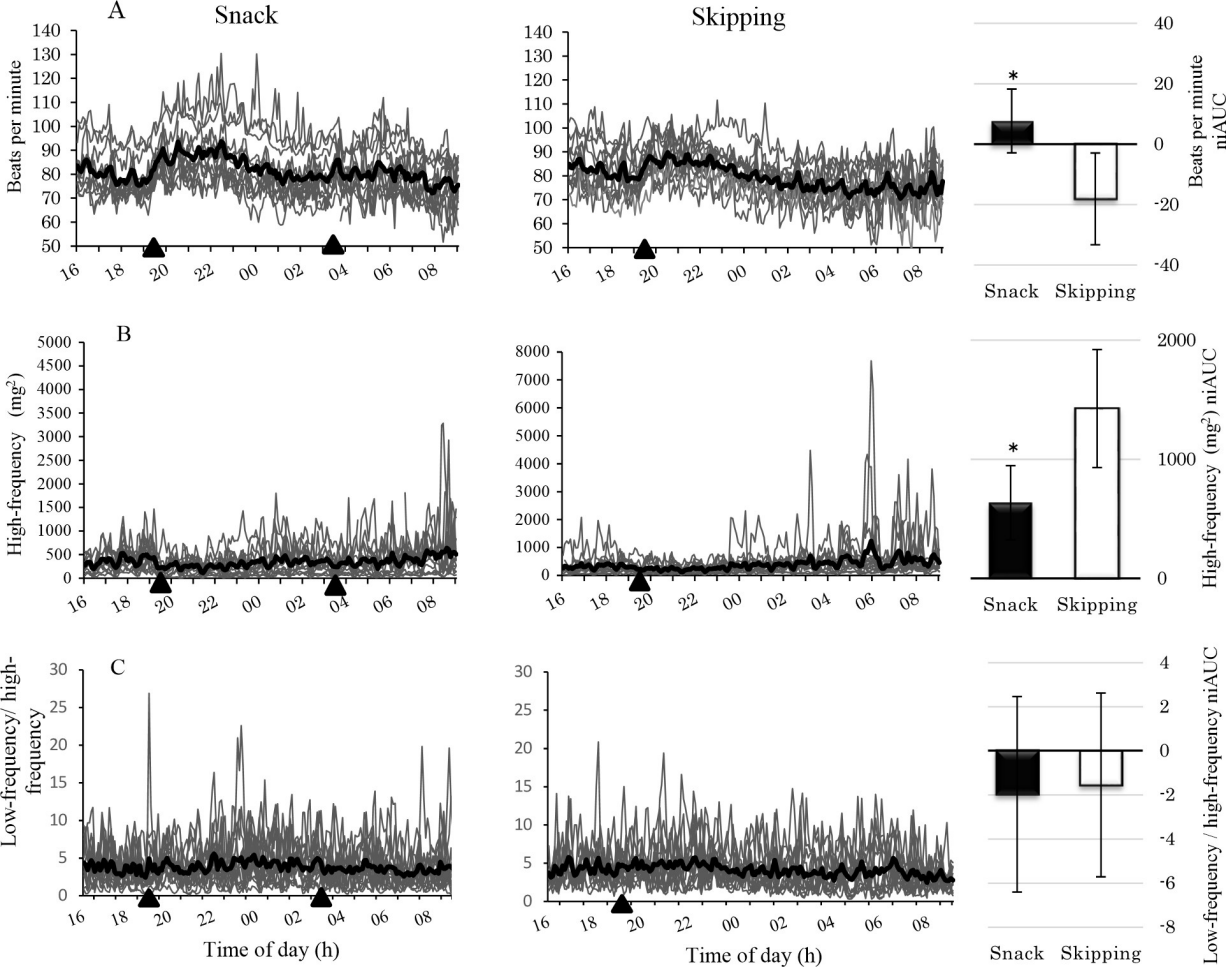
Heart rate, high-frequency, and low-frequency/high-frequency ratio following a simulated night shift. A-HR, B-HF. C-LF/HF, Left side and center panels display the individual data per condition from 16:00 to 09:00 testing points. The mean is shown as a thicker line over the top of the individual data. Snack condition—left side panel, Skipping condition—center panel. Black triangle (▲) indicates eating a meal at 19:30 and at 03:30. The average values of the markers in the left side and center panels match columns in the right side panels for each condition, before and after eating, from 03:00 to 09:00. Bars represent standard error. Analysis of covariance was used to assess the significance of mean differences between groups after eating (*: p < .05).

Electrocardiogram data could not be obtained from one participant during the experiment; therefore, data were analyzed for 14 participants. Fig 5A shows the heart rate data. Significant main effects of condition (*p* < .001) and their interaction were observed (*p* = .029). Under the Snack condition, heart rate increased significantly at 04:00 (*p* = .028), 05:00 (*p* = .046), and 06:00 (*p* = .028) compared with the Skipping condition, and showed an increasing tendency at 07:00 (*p* = .078).

A significant main effect of condition was observed for high-frequency power (Fig 5B), which tended to be significantly lower under the Snack condition than under the Skipping condition between 04:00 and 09:00 (*p* = .001). In addition, there was a significant main effect of time (*p* = .007), and a marginally significant condition * time interaction (*p* = .081) was seen for the low-frequency/high-frequency ratio (Fig 5C). In the Snack condition, the low-frequency/high-frequency ratio was significantly lower at 03:00 (*p* = .041), 04:00 (*p* = .038), 06:00 (*p* < .001), 07:00 (*p* < .001), and 08:00 (*p* < .001) after eating than at 02:00 before eating.

### Performance and temperature and autonomic nervous system parameters

**Fig 6:**
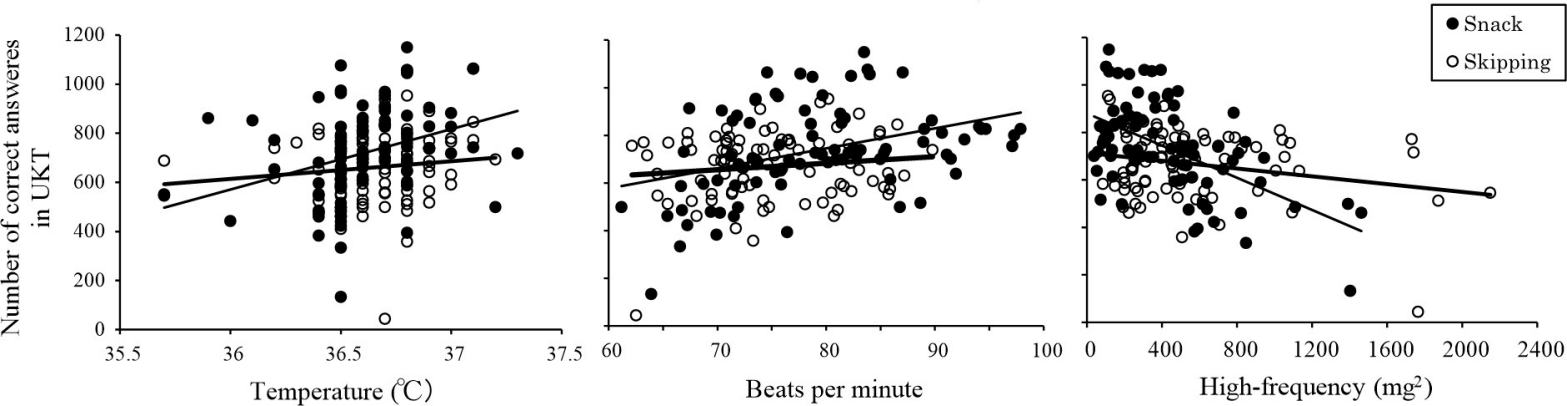
**Scatterplots for change on the Uchida-Kraepelin Test after eating (04:00 to 09:00) compared to temperature (left panel), heart rate (center panel), and high-frequency after eating (right panel).** Trend lines are shown for each condition (Snack: black circles, solid lines; Skipping: white circles, dashed lines).

Table 3 displays the results of the correlational analyses of temperature and autonomic nervous system parameters with performance (PVT and UKT). Significant relationships were found on the UKT for temperature, HR, and HF in the Snack condition, with higher temperature (r = 0.430, *p* < .001), higher HR (r = 0.404, *p* < .001), and less HF (r = -0.478, *p* < .001) (Fig 6).

**Table 3:** Correlations of after eating (04:00 to 09:00) performance outcomes with temperature and autonomic nervous system parameters.

	PVT		UKT	
Variable	Snack	Skipping	Snack	Skipping
Temperature	0.093	—0.084	**0.430**	0.146
HR	0.013	—0.007	**0.404**	0.057
HF	0.110	0.141	**—0.478**	—0.110
LF/HF	—0.097	0.122	0.138	0.094

Significant correlations are highlighted in bold (p < .001).

HR: Heart rate; HF: high-frequency power; LF/HF: low-frequency power/high-frequency power ratio.

## Discussion

The present study investigated the effects of nighttime eating during night shifts on temperature, subjective sleepiness, fatigue, hunger, vigilant attention, processing speed, and autonomic nervous system activity during testing between 16:00 and 09:00. Increasing perceptions of hunger across night shifts have been reported by shift workers, who have described hunger as a factor that influences their decision to eat during the night shift [8]. In the present study, consuming a snack (352 kcal) during the night reduced perceptions of hunger, maintained performance, and increased heart rate for 3 h. Furthermore, the snack likely increased body temperature (i.e. food-induced thermogenesis [33]), which could in turn increase alertness. To the best of our knowledge, this study is the first to investigate each hour the effects of consuming a snack on objective nighttime sleepiness and fatigue, vigilant attention, information processing, and changes in autonomic nervous system activity over a simulated night shift in a controlled laboratory environment.

Regardless of the Snack condition, the greatest impairments in subjective sleepiness and fatigue, vigilant attention, and performance increased toward morning. This could explain the increases in circadian sleep pressure and decreased alertness throughout the night [34]. Consistent with our previous study [35], although sleepiness and fatigue increased across the simulated 16-h night shift, no differences were found between participants who had and had not eaten during the night [12, 36]. Whereas the results after 04:00 showed increased subjective sleepiness and fatigue [37], this might correspond with the circadian low points between 02:00 and 06:00 [34]. Previous studies have suggested that snacking (10% 24-h estimated energy requirement) at 00:30 did not increase sleepiness at 02:30 and 05:00 [10], but eating at 01:30 is not a suitable mealtime, because sleepiness increased at 03:00. In the present study, sleepiness and fatigue did not differ, regardless of eating. It is speculated that rice balls are mainly carbohydrates, low in fat, and relatively slow to digest and absorb, and their granular shape is presumed to require chewing; in addition to the effect on motor control, chewing increases the arousal level and alertness [38]. Thus, these factors may have prevented a significant increase in drowsiness [38]. Therefore, we concluded that the ingestion of the snack (352 kcal) did not change the subjects’ sleepiness and fatigue. Performance on the PVT reflects circadian modulations in neurobehavioral functions, in addition to the effects of sleep pressure that develop with an increased duration of waking time, without being confounded by a learning curve [39]. PVT, mean RT, and lapses were similar between the two conditions. Subjective sleepiness is more likely to be underestimated [39] than objectively evaluated sleepiness [40]. Drowsiness weakens alertness and is likely to cause human error. In this study, the snack at 03:30 was affected by circadian modulations in neurobehavioral functions that developed in association with waking time and sleep pressure; decreases were seen in attention, but the PVT false starts were significantly higher in the Skipping condition than in the Snack condition. In particular, night shift workers are prone to errors at dawn, but it has been shown that light meals may make it possible to prevent accidents in the morning.

According to recent reviews, eating breakfast may result in acute improvements of memory, attention, and motor and executive function, although no conclusion about the effect of macronutrients on cognitive function has been reached [38]. The present result shows that a small snack lessened the decline in the number of correct answers on the UKT. The UKT, which is used to measure cognitive task performance, involves simple mental arithmetic and handwriting [38]. Several cognitive domains, including sustained attention and short-term memory, are involved in the mental arithmetic task. Handwriting is also a complex perceptual–motor skill [40]. Correlations among PVT, UKT, temperature, and autonomic nervous system parameters showed that, in the Snack condition, both increased temperature and HR and decreased HF were observed. The UKT is a more complex task than the PVT, so a difference was observed. When performing complex tasks, the brain regions involved in sustained attention and short-term memory are active separately with temporal fluctuations. Therefore, even if the function of the part responsible for the ability to sustain attention deteriorates due to lack of sleep, the brain parts responsible for each function may complement each other and not be reflected in the task performance (i.e. masking effect). It is thought that the more complex tasks, the more likely the mutual complementation of cerebral functions will have the effect of counteracting sleep deprivation [41]. We think that a small snack in the night, as do daytime meals, can increase body temperature, which can in turn increase alertness, and be complementary and maintain brain functions during a night shift.

Given recent studies highlighting the potential metabolic consequences of consuming a large nighttime meal [42, 43], a small meal, such as two rice balls, may be an option that is more readily available to maintain the cognitive function of shift workers.

These findings indicate that the subjects who ate a small meal felt less hungry for 5 h (i.e., until 09:00), and that those who did not eat at night perceived greater hunger toward morning. Although these findings may predominantly reflect a longer inter-meal interval, hunger is known to display a circadian rhythm and be increased during the night. Restricted sleep also increases hunger and appetite, and it results in a preference for a high-energy diet rich in carbohydrates [15]. Night work has been reported to cause mental stress [44], which can trigger fatigue and lead to decreased performance [45, 46]. As short-term changes in food intake are known to affect various aspects of cognitive function, we believe that reducing the stress induced by hunger may help sustain performance. Compared with high-fat meals, those rich in carbohydrates reduce mental (in contrast to physical) performance and increase sleepiness [47]. Eating a heavy lunch is associated with significant increases in motor vehicle accidents and a tendency to experience greater subjective sleepiness [48]. More generally, objective signs of sleepiness typically peak about 3.5 h after eating [49]. In other words, in this respect, tasks that require sustained attention are the most sensitive, with larger meals producing more frequent lapses of attention [50]. However, in the present study, the consumption of two rice balls (containing approximately 352 kcal of primarily carbohydrates) did not appear to affect sleepiness and fatigue.

In the present study, the heart rate was significantly higher under the Snack condition than under the Skipping condition from 04:00 to 06:00. When eating a meal, visceral circulating blood volume increases to support the process of digestion and absorption, the sympathetic nervous system supports it, and the heart rate increases [51]. The contribution of increased sympathetic nervous system activity postprandially to the thermic effect of food is not always evident, and it has been shown to depend on the size and composition of the meal, with the clearest effect seen with carbohydrates. The brain integrates signals related to food intake from various sites (e.g., gut, hepatoportal area, chemoreceptors), leading to increased peripheral sympathetic outflow [51]. Few studies have focused on the relationship between meals and autonomic nervous system activity. The results of the present study showed that a small meal increased the heart rate and decreased parasympathetic nervous system activity, which is related to rest and digestive activity. The two systems, the sympathetic nervous system and the parasympathetic nervous system, generally act in a complementary fashion, because an increase in one is usually associated with a decrease in the other. Many sympathetic nervous system functions are opposite to those of the parasympathetic nervous system. In the present results for the low-frequency power/high-frequency power ratio values between 02:00 and 09:00, the Skipping condition showed no change, but the Snack condition resulted in an immediate decrease after eating. Because the low-frequency power/high-frequency power ratio has been shown to represent changes in sympathetic nervous activity in the autonomic nervous system as a stress index [52], stress levels may have been lower under the eating condition. Consuming a meal is reportedly effective for reducing stress [53], which is consistent with the results of the present study. Among night shift nurses who remain awake for a long period of time, sleepiness and fatigue increase, and stress levels also rise gradually with increased workloads around the end of their shifts [54]. If stressful situations are prolonged, night shift workers may develop chronic fatigue. The results of this study suggest that eating a small meal at 03:30 may be effective for reducing stress in the morning. In addition, shift workers often report that increased alertness is one of the main factors that influences their decision to eat during a shift [12]. Dietary strategies and altered eating behaviors have been reported as strategies to maintain alertness during the night shift among a sample of nurses [55]. Furthermore, reduced meal sizes have been reported as a strategy used by nurses to minimize gastric symptoms during night shifts. Therefore, promoting the consumption of a small meal at 03:30 during break time may help night shift workers maintain their health and improve safety during 16-h night shifts, as well as prevent malpractice and chronic fatigue.

In the case of long night shifts, the time for taking meals is restricted, and it is difficult to eat freely. We recommend a small snack to maintain performance and stop the increase in errors regardless of time. Rice balls, which can be easily eaten even when busy, may be one of the options for meals during night shifts.

### Limitations

This study has several limitations. First, it was conducted under laboratory conditions; therefore, the degree of change in performance and sleepiness could vary from that under actual working conditions, depending greatly on differences in workload due to the nature of the work or the timing of busy work periods. To confirm effective measures for reducing sleepiness and fatigue tailored to the conditions of specific professions and workplaces, intervention studies need to be conducted in actual workplaces. It is possible that the present results would be different if actual shift workers had been studied; therefore, caution is needed when interpreting the results. Second, the data were obtained under the condition that everyone was served the same meal at the same time, and that meals were standardized and not designed for each individual. It is plausible that variability in meal timing (masked by social jet lag) could be triggering the misalignment between peripheral and central clocks [56]. Third, the participants in this study were all young women in their 20s with no shift work experience, which may have affected the results. We would like to investigate this issue further in a future study.

A previous study reported that taking a nap during a night shift helped nurses recover from night shift fatigue [57]. Therefore, in the future, it will be necessary to take measures to reduce drowsiness and fatigue by combining meals and naps.

## Conclusions

This study investigated the effects of eating a meal at night during night shifts on subjective sleepiness, vigilant attention, processing speed, hunger, and autonomic nervous system activity between 16:00 and 09:00. The results showed that consuming a small meal (352 kcal) at 03:30 during the night shift reduced hunger and helped sustain performance until morning. To the best of our knowledge, this is the first study to explore the effects of consuming a small meal at night on 16-h performance in a controlled laboratory environment.

## Supporting information

S1 Checklist CONSORT checklist. (PDF)

S1 Protocol Study protocol. https://upload.umin.ac.jp/cgi-open-bin/ctr/ctr_view.cgi?recptno=R000039151.
(PDF)

S1 Appendix The minimal data set collected from the study population. (CSV)

## Abbreviations

PVT: Psychomotor vigilance test
HRV: Heart rate variability
UKT: Uchida-Kraepelin Test
SD: Standard deviation
HR: Heart rate
LF/HF: Low-frequency/High-frequency
HF: High-frequency
