# Supplementary material for: Effects of a snack on performance and errors during a simulated 16-h night shift: A randomized, crossover-controlled, pilot study
Source: PLoS One. 2021 Oct 22;16(10):e0258569. doi: 10.1371/journal.pone.0258569 (PMC8535457; doi:10.1371/journal.pone.0258569)
Supplement: S1 Protocol — https://upload.umin.ac.jp/cgi-open-bin/ctr/ctr_view.cgi?recptno=R000039151. (PDF) [file pone.0258569.s002.pdf]

The main points of clinical study protocol (Translation from Japanese to English)

Title of Study

Effects of a snack on performance and errors during a simulated 16-h night shift: A randomized crossover-controlled trial

Research contact person: Sanae Oriyama
Organization: Hiroshima University
Division: Nursing Science
Address: 1-2-3, Kasumi, Minami-ku, Hiroshima
Tel: 81-82-257-5355
E-mail: oriyama@hiroshima-u.ac.jp

Research Institute: Hiroshima University

Ethical Review Committee: the Center for Integrated Medical Research of Hiroshima University

Approved Date: 1, October, 2018.

Synopsis

Study Protocol and Ethical Consideration

Study objective	To examine the effect of a snack on performance and errors during a simulated 16-h night shift.
Study Design	Randomized controlled crossover trial
Participants	1. Female university students aged 20-23 years old.<br>2. Women with neither morning type nor evening type.<br>3. Women who joined in the explanation session and gave written informed consent.
Target sample size	15
Exclusion criteria	1. Patient with sleep disorder<br>2. The participants were not obvious morning type or evening type.
Groups	Participants are randomly allocated to two groups (Sequence A, B)
Intervention	Two nights interventions are conducted in the following order. There are wash-out periods (4 weeks) between each intervention period.<br>Sequence A 1st: no snack at 03:30 2nd: snack at 03:30<br>Sequence B 1st: snack at 03:30 2nd: no snack at 03:30
Primary outcomes	Uchida-Kraepelin Test (UKT)
Secondary outcomes	Sublingual temperature<br>Psychomotor vigilance test (PVT)<br>Autonomic nervous system activity<br>Subjective assessment of sleepiness, fatigue, and hunger (VAS)
Informed consent	Before participating the study, the potential participants must join study information session with sufficient detail including the purpose, the method and ethical consideration and, voluntarily sign the informed consent format. Further, the participants are guaranteed the right to withdraw their consent after entering the study.
Data Management	The anonymous data with identification number excluding names, addresses or telephone numbers are stored in the locked cabinet for five years after this study finished.

Consultation from participants	If the participants hope to ask any questions or withdraw their consent, they can contact the authors by referring the telephone number, e-mail address in the handout.
Publication	The findings of this study will be published in publications or presented at scientific meetings.
Financial Disclosure	This study is supported by JSPS KAKENHI, Grant number 26293452
Conflict of Interest	The authors declare that no conflict of interests exists.
Planned follow-up period	2018 autumn
